# Bio-prospecting of cuttle fish waste and cow dung for the production of fibrinolytic enzyme from *Bacillus cereus* IND5 in solid state fermentation

**DOI:** 10.1007/s13205-016-0553-0

**Published:** 2016-10-28

**Authors:** Gurupatham Devadhasan Biji, Arumugaperumal Arun, Eswaran Muthulakshmi, Ponnuswamy Vijayaraghavan, Mariadhas Valan Arasu, Naif Abdullah Al-Dhabi

**Affiliations:** 1 Department of Zoology, Nesamony Memorial Christian College, Marthandam, Kanyakumari, Tamil Nadu 629 165 India; 2 Department of Biotechnology, Kalasalingam University, Virudhunagar, Srivilliputtur, Tamilnadu 626 126 India; 3 Centre for Marine Science and Technology, Manonmaniam Sundaranar University, Rajakkamangalam, Kanyakumari, Tamil Nadu 629 502 India; 4 Department of Botany and Microbiology, Addiriyah Chair for Environmental Studies, College of Science, King Saud University, P. O. Box 2455, Riyadh, 11451 Saudi Arabia

**Keywords:** Cuttle fish waste, Cow dung, Solid state fermentation, Fibrinolytic enzyme, *Bacillus cereus* IND5, Thrombolytic therapy

## Abstract

The process parameters governing the production of fibrinolytic enzyme in solid state fermentation employing *Bacillus cereus* IND5 and using cuttle fish waste and cow dung substrate were optimized. The pH value of the medium, moisture content, sucrose, casein and magnesium sulfate were considered for two-level full factorial design and pH, casein and magnesium sulfate were identified as the important factors for fibrinolytic enzyme production. Central composite design was applied to investigate the interactive effect among variables (pH, casein and magnesium sulfate) and response surface plots were created to find the pinnacle of process response. The optimized levels of factors were pH 7.8, 1.1% casein and 0.1% magnesium sulfate. Enzyme production was increased 2.5-fold after statistical approach. The enzyme was purified up to a specific activity of 364.5 U/g proteins and its molecular weight was 47 kDa. It was stable at pH 8.0 and was highly active at 50 °C. The mixture of cuttle fish waste and cow dung could find great application as solid substrate for the production of fibrinolytic enzyme.

## Introduction

Cardiovascular diseases (CVDs) namely, coronary heart disease, acute myocardial infarction and atherosclerosis are the leading cause of death. Among these CVDs, thrombosis is one of the most important diseases (Wang et al. 2006). CVDs are treated by the extensive use of tissue-type plasminogen activator (t-PA), urokinase and streptokinase among other fibrinolytic agents (Holden 1990). However, these fibrinolytic agents cause allergic reactions, bleeding complications and short half-lives (Blann et al. 2002; Bode et al. 1996; Turpie 2002). Hence, the search is still vibrant for new and safer fibrinolytic enzymes throughout the world. In recent years, microbial fibrinolytic enzymes have been considered cost effective among the other fibrinolytic enzymes. These enzymes have been extensively studied from insects (Ahn et al. 2003), snake venom (He et al. 2007), and marine organisms (Sumi et al. 1992). Fermented foods namely, natto (Sumi et al. 1987, 1990; Fujita et al. 1993, 1995), Chungkook-Jang (Kim et al. 1996) and Tempeh (Sugimoto et al. 2007) have also been the source of fibrinolytic enzyme isolation. Apart from therapeutic applications, fibrinolytic proteases continue to be attractive as their potent activity on blood clot (He et al. 2007), Keratin (Bressollier et al. 1999) and collagen (Itoi et al. 2006) could have important industrial waste management and medical applications.

Solid state fermentation (SSF) is emerging as an efficient technique for production of enzymes and bioconversion of metabolites. Agro-industrial wastes, waste water and fishery wastes were utilized as cost-effective substrates in processes of biomolecule production. The agro-wastes namely, green gram husk (Prakasham et al. 2006), cake of *Jatropha curcas* seed (Mahanta et al. 2008), ground nut husk (Salihu et al. 2014), copra waste (Dilipkumar et al. 2013) and wheat bran and deproteinized acid cheese whey (Raol et al. 2015) were used successfully as the substrate for the enzyme production. The ideal substrate should be cheap so as to reduce the production cost of biomolecules (Pandey et al. 2000). In SSF process, the solid substrate provides required nutrient to the growth of microbes and enzyme production. Hence, the solid substrate employed for any bioprocess should be rich in nutrients for the production of biomolecules. Recently, there is an increase in the exploitation of resources from marine wastes. About 105.6 million tons of fish resources were utilized for human consumption and 34.8 million tons (25% of the fish resource) are treated as waste. The fishery waste includes whole fish waste, viscera, bones, skin, fish head, gonads, frame liver, muscle tissue and other parts (Awarenet 2004). However, these wastes were left unused or buried along sea shore which causes pollution to the environment (Bozzano and Sardà 2002). Several studies were carried out to utilize these wastes as substrate for enzyme production. The fish wastes included fish meat wastes (Vázquez et al. 2006), head and viscera powder (Triki-Ellouz et al. 2003) and waste water (Haddar et al. 2010). These fish wastes were rich of proteins, amino acids and oils (Ghaly et al. 2013). On the other hand, cow dung is one of the cheapest biomass which proved to be a useful substrate in protease (Vijayaraghavan and Vincent 2012), cellulase (Vijayaraghavan et al. 2016a) and fibrinolytic enzyme (Vijayaraghavan et al. 2016b) production in SSF. It contains cellulose, carbon, hemicelluloses, ions and trace elements (Fulhage 2000) and it could be a novel feedstock for better growth of microorganisms (Adegunloye et al. 2007). Considering the nutritive value of fish waste and cow dung, this combination of feedstock was used as the solid substrate for fibrinolytic enzyme production.

Statistical designs of experiments help to find the important variable and concentration of variables for biomolecules production. These were traditionally evaluated by one–factor–at–a–time strategy. However, it fails most often to nail down the correct response in an enzyme bioprocess and this can be overcome by statistical experimental design. The statistical designs such as fractional factorial design (Liu et al. 2005), Plackett–Burman (Dilipkumar et al. 2011) and L_18_-orthogonal array (Mahajan et al. 2012) were used to screen the significant variables affecting biomolecule production. Response surface methodology (RSM) was used to find the optimum concentration of these important variables, and thereby used to enhance the production of acid proteases (Siala et al. 2012), fibrinolytic enzymes (Vijayaraghavan and Vincent 2014) and β-galactosidase (Raol et al. 2015). In this study, optimization of medium components involved in fibrinolytic enzyme production by *Bacillus cereus* IND5 using fish waste and cow dung substrate was carried out using RSM. Based on our knowledge, no work is available regarding fibrinolytic enzyme production using the mixture of these feedstock (fish waste and cow dung) in solid state fermentation. Hence, this substrate was selected for the production of fibrinolytic enzyme.

## Materials and methods

### Screening of organism producing fibrinolytic enzyme

About 1 g fermented rice was mixed with 100 ml distilled water. Sample was taken from it and screened for organisms showing proteolytic activity using skimmed milk agar plates. Ten bacterial cultures, which showed clear zone in the casein agar plates, were further screened for fibrinolytic enzyme producing ability. The protease positive isolates were cultured in a medium containing peptone (3.0%), glucose (1.0%), CaCl_2_ (0.50%), and MgSO_4_ (0.20%). The pH of culture medium was brought to 7.0, and incubated at 37 °C for 72 h at 150 rpm (Gad et al. 2014). After 72 h of incubation, the bacterial biomass was separated by centrifugation (8000 rpm, 10 min, and 4 °C) and the extract devoid of cells was used to screen fibrinolytic enzyme activity. Fibrin plate assay was used to assess the fibrinolytic activity of the extracts. The fibrinolytic protease activity appeared as a halo zone around the fibrin clot after incubation at 37 °C for a period of 5 h.

### Identification of the bacterial isolate

A bacterial isolate (IND5), which showed good activity based on the above screening procedures, was further identified using biochemical and morphological tests (Bednarski 2006). The 16S rRNA sequencing was carried out using the forward (5′AGAGTTTGATCMTGGCTAG3′) and reverse primer (5′ACGGGCGGTGTGTRC3′) (Rejiniemon et al. 2015). Amplification of 16S rRNA gene was carried out using a gradient PCR machine (Peng et al. 2004). The available sequence was compared using NCBI BLAST and the organism was identified as *Bacillus cereus* IND5.

### Solid state fermentation (SSF)

Cuttle fish was collected from Kanniyakumari coast and its by-product was prepared (Souissi et al. 2008). Briefly, gut, stomach and head were removed, rinsed with double distilled water, heated, minced and dried well at 80 ± 2 °C for 48 h. Cow dung substrate was processed as described previously (Vijayaraghavan et al. 2012). Equal amount of cuttle fish and cow dung waste were mixed and used as the substrate. 5 g of substrate (2.5 g cuttle fish waste +2.5 g cow dung) was taken in 100 ml Erlenmeyer flasks and the pH of the solid substrate medium was adjusted to 8.0 by the addition of 0.1 M tris–HCl buffer. The moisture content of SSF medium was adjusted to 70% (v/w). During the preliminary experiments, the Erlenmeyer flasks were incubated at 37 °C for 72 h, and at 96 h, the maximum production of fibrinolytic enzyme was registered. Hence, all fermentations were run for a period of 72 h.

### Submerged fermentation

In the present study, *B. cereus* IND5 was cultured in submerged fermentation to compare the yield with that of SSF. For this, 100 ml nutrient broth medium (beef extract, 5 g/l; peptone, 5 g/l; yeast extract, 1.5 g/l; and sodium chloride) was prepared, sterilized and inoculated with 0.1 ml of 18 h grown *B. cereus* IND5. The Erlenmeyer flask was incubated at 37 °C for 48 h. The culture was centrifuged at 10,000 rpm for 10 min and the cell free supernatant was used as the crude enzyme.

### Fibrinolytic enzyme assay

The enzyme (0.1 ml) was mixed with 2.5 ml of tris–HCl buffer (pH 7.8) containing calcium chloride (0.01 M). Fibrinolytic activity was assayed on fibrin substrate. The absorbance was read at 275 nm (Anson 1938; Chang et al. 2000). Enzyme activity was calculated based on the calibration curve drawn for standard solution of l-Tyrosine. One unit of fibrinolytic enzyme activity (U) was defined as the amount of enzyme which liberates 1 µg of tyrosine per min under the standard assay condition. The results of the determination of fibrinolytic activity were described in units of activity/gram of substrate (U/g).

### Selection of important medium components for fibrinolytic enzyme production by one variable at a time approach

In the present study, the mixture of cuttle fish waste and cow dung was used as the substrate for the optimization of enzyme production. The effect of six different carbon sources namely, glucose, starch, trehalose, xylose, sucrose, and maltose on the production of fibrinolytic enzyme was studied. To evaluate the influence of nitrogen sources, beef extract, casein, gelatin, urea, and yeast extract were employed. The solid medium was supplemented with ammonium chloride, ammonium sulfate, calcium chloride, di-sodium hydrogen phosphate (Na_2_HPO_4_), ferrous sulfate, sodium di-hydrogen phosphate (NaH_2_PO_4_), and sodium nitrate, to evaluate the influence of ions on production of enzyme. These nutrients were supplemented with the substrate individually. The substrate was mixed carefully with tris HCl buffer (pH 8.0) to adjust the initial moisture to 70%. Then, 500 µl inoculum (10%, v/w) was introduced into the medium and incubated as described previously. Fifty milliliter of ice cold double distilled water was poured with the fermented medium and shaken at 150 rpm for 30 min. It was further centrifuged at a speed of 10,000 rpm for 20 min in a centrifuge maintained at 4 °C. The cell free extract was stored and utilized for fibrinolytic enzyme assay.

### Screening of vital medium components using two-level full factorial design

A two-level full factorial statistical design, a preliminary medium optimization strategy to find the important medium components in fibrinolytic enzyme production, was employed in this study. For screening of vital medium components, five variables, pH, moisture, sucrose, casein and MgSO_4_ were selected. In two-level full factorial design, each variable is represented at the low (−) level and high (+) level. The design model is based on the first order polynomial equation:

1 $$Y = \alpha_{0} + \mathop \sum \limits_{i} \alpha_{i} x_{i} + \mathop \sum \limits_{ij} \alpha_{ij} x_{i} x_{j} + \mathop \sum \limits_{ijk} \alpha_{ijk} x_{i} x_{j} x_{k} ,$$

where *α*_0_, *α*_*i*_, *α*_*ij*_ and *α*_*ijk*_ represent the intercept, *i*th linear coefficient, *ij*th interaction coefficient and the *ijk*th interaction coefficient, respectively. SSF process was initiated in 100 ml Erlenmeyer flasks and the same were maintained at 37 °C for 72 h using an incubator. Each experiment was carried out in duplicate and average value was presented. After statistical analysis using analysis of variance (ANOVA), the important factors were identified. Design-Expert 9.0 (StatEase Inc, Minneapolis, USA) was sought for the design of experiments and statistical analysis of data.

### Response surface methodology

The significant medium ingredients (pH, casein, and MgSO_4_) affecting fibrinolytic enzyme production, as observed by the two-level full factorial design, were tested for interactive effects using a central composite design (CCD) of response surface methodology. The selected variables were coded as A (pH), B (casein), and C (MgSO_4_) and the following second order model equation was used to predict the response (Eq. 2).

2 $$Y = \beta_{0} + \mathop \sum \limits_{i = 1}^{3} \beta_{i} X_{i} + \mathop \sum \limits_{i = 1}^{3} \beta_{ii} X_{i}^{2} + \mathop \sum \limits_{ij = 1}^{3} \beta_{ij} X_{ij} .$$

The experimental runs were performed in 100 ml Erlenmeyer flasks as per the central composite design in randomized manner. The substrate was sterilized at 121 °C for 20 min and cooled. After which, 10% inoculum (*B. cereus* IND5) were carefully inoculated under aseptic conditions. SSF was carried out in an incubator maintained at 37 °C for a period of 72 h. After fermentation, enzyme in the SSF medium was extracted and enzyme assay was carried out. The ANOVA was used to evaluate the significant model terms. The optimum medium composition was obtained using RSM and these optimum conditions were validated. Design-Expert 9.0 (StatEase Inc, Minneapolis, USA) was the software program employed to design the experiment and to analyze the data.

### Purification of fibrinolytic enzyme from *B*. *cereus* IND5

The fibrinolytic enzyme was produced through SSF using *B. cereus* IND5 using the optimized culture medium. The crude enzyme was purified by performing ammonium sulfate precipitation, diethyl aminoethyl cellulose (DEAE cellulose) and casein-agarose affinity chromatography. 50 ml of crude extract was precipitated and 70% saturation was attained by the addition of ammonium sulfate salt. The precipitate was separated out by centrifugation (10,000 rpm, 4 °C) and was dissolved in 0.1 M tris–HCl buffer (pH 8.0). It was further dialyzed against water (two changes) and buffer (third change). The sample was further loaded on a DEAE-cellulose column which was pre equilibrated with 0.05 M tris–HCl buffer (pH 8.0). The bounded proteins were eluted with 0.05 M tris buffer containing 0–1.0 M NaCl. NaCl gradient was made using a gradient maker and all fractions were collected manually. The fractions (2.0 ml) were analyzed for their protein concentration (Absorbance 280 nm) and enzyme activity. The active fractions were loaded on casein-agarose affinity chromatography (Sigma, USA) which was pre equilibrated with 0.02 M tris–HCl buffer. Elution of the proteins was done with a gradient of buffer with strength of 0–0.8 M NaCl. The fractions resulting from elution were assayed for fibrinolytic activity. In each step of enzyme purification, enzyme assay and also total protein estimation were carried out. Further, specific activity of enzyme, yield, and purification fold were also measured.

### SDS-PAGE analysis and zymography

The highly active fraction from DEAE cellulose chromatography and affinity chromatography was subjected to analysis for homogeneity. The enzyme sample was added with SDS sample buffer and boiled for 1 min before loading on to the SDS-PAGE gel (12%). The molecular markers used were soybean trypsin inhibitor of 20.1 kDa, carbonic anhydrase of 29 kDa, ovalbumin of 43 kDa, bovine serum albumin of 66 kDa and phosphorylase b of 97.4 kDa. The protein bands were visualized by staining using coomassie brilliant blue (R-250). For zymography analysis, a fibrin substrate containing polyacrylamide gel (12%) was prepared by adding 0.12% (w/v) fibrinogen and thrombin (100 NIH U/ml). The affinity chromatography purified-fraction was loaded on this gel. At the end of electrophoresis, the gel was taken out and incubated with buffer A containing 2.5% (v/v) Triton X-100 for 30 min at room temperature (30 ± 1 °C). The residual Triton X-100 was removed by washing the gel with double distilled water for 30 min. The gel was incubated with buffer A for 4 h. Then the gel was subjected to coomassie brilliant blue (R-250) staining for 2 h and then destained (overnight). The fibrinolytic activity of the enzyme was visualized as nonstained region on blue background.

### Evaluation of characteristics of the purified enzyme

The optimal pH needed for the maximized fibrinolytic activity of enzyme, was estimated using buffers (0.1 M) of different pH namely, pH 3.0 and 4.0 (citrate buffer), pH 5.0 (succinate buffer), pH 6.0 and 7.0 (sodium phosphate buffer), pH 8.0 (tris buffer) and pH 9.0 and 10.0 (glycine-NaOH buffer). To evaluate the stability of enzyme activity with respect to pH, the fibrinolytic enzyme was incubated with the aforementioned buffers, separately and incubated at 37 °C for 1 h before adding the substrate. The influence of temperature on the activity of enzyme was estimated by performing the reactions at different temperatures of 30, 40, 50, 60, and 70 °C. The stability of the fibrinolytic enzyme with respect to temperature was estimated by incubating it in the absence of substrate at different temperatures, 30–70 °C for 1 h. Fibrinolytic activity of the enzyme was assayed as described earlier. To elucidate the influence of divalent ions on enzyme activity, the enzyme was incubated for 30 min along with different divalent ions, namely Ca^2+^, Cu^2+^, Co^2+^, Mg^2+^, Mn^2+^, Hg^2+^, Fe^2+^, and Zn^2+^ at 0.01 M concentrations (Lomate and Hivrale 2011).

## Results and discussion

### Screening and identification of the fibrinolytic enzyme producing bacterium

**Fig. 1:**
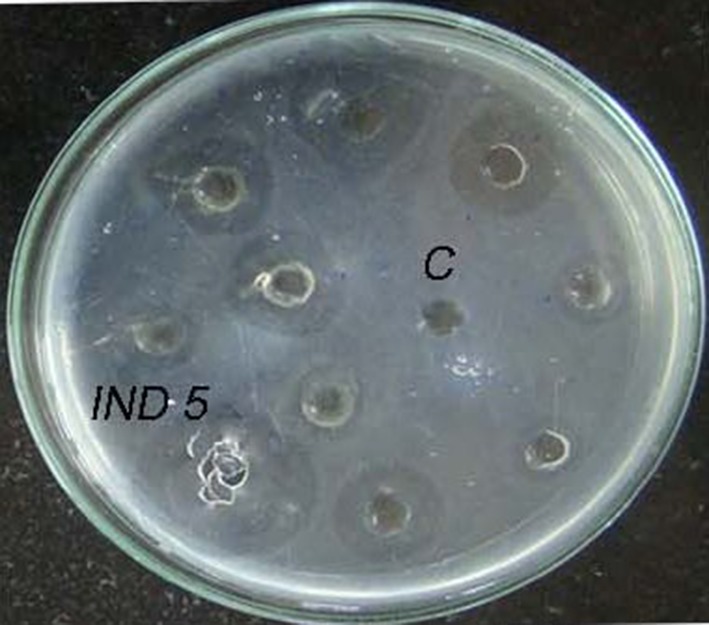
Fibrinolytic enzyme activity of the bacterial isolates on fibrin plate (C-control)

Ten protease producing bacterial isolates were selected after casein hydrolysis. These protease positive isolates were further evaluated for production of fibrinolytic enzyme using fibrin plate. Among the ten bacterial cultures, the cell free extract of an isolate named IND5 produced the highest halo zone than other isolates (Fig. 1). The halo zone represents the clearance of fibrin which in turn indicates the fibrinolytic activity of the strains. The isolate IND5 also produced more fibrinolytic enzyme in submerged fermentation compared to the other isolates. The 16S rDNA sequence of the isolate IND5 was subjected to BLAST and the sequence showed a high similarity with *Bacillus cereus*. The strain was identified as *B*. *cereus* IND5 and accession number was assigned (KF250421).

### Production of fibrinolytic enzyme in submerged fermentation

*B*. *cereus* IND5 was cultured in nutrient broth medium and enzyme production was found to be 73 U/ml after 48 h of incubation at 37 °C. Submerged fermentation has been widely used for the production of enzymes. Recently, Bajaj et al. (2014) used submerged fermentation for the production of fibrinolytic enzyme from *Bacillus* sp.

### Cuttle fish waste and cow dung mixture for fibrinolytic enzyme production in SSF

In SSF, enzyme production was found to be maximum after 72 h incubation at 37 °C (1205 ± 48 U/g). SSF technique has been widely used for the production of enzymes, antibiotics, secondary metabolites, flavoring compounds and also animal feeds. SSF process is defined as the one in which microbes get attached to solid materials and grow, without any free water. In SSF process, the solid substrate provides anchorage for the cells and supply nutrients. Hence, the native-like environment is created for the organism and maximum product production is expected. In recent years, many substrates were mixed and used as the fermentation medium to confirm its balanced nutritive value. The mixture of substrates, such as, protein and chitin (Wang et al. 2008b), powdered crab and shrimp shell (Oh et al. 2000), cuttlefish by-products powder and wastewater from fish processing industry (Souissi et al. 2008), cuttlefish and shrimp by-products (Ben Rebah et al. 2008) were used for the production of various enzymes. The by-products from fish processing support growth of microbes in an excellent manner. Due to its low cost and easily available nature, these substrates play a significant role in the production of enzyme (Rebah and Miled 2013). Mukherjee et al. (2008) used a mixture of potato peel with Imperata cylindrica grass as the substrate and produced fibrinolytic enzyme. Cow dung was reported as a novel substrate for use in SSF aimed at the production of cellulases (Vijayaraghavan et al. 2016a) and fibrinolytic enzymes (Vijayaraghavan et al. 2016b). Ghorbel et al. (2005) stated the importance of balanced nutrients for enzyme production by microbes. Considering the importance of balanced diet, the combination of cuttlefish by-product and cow dung was used as a substrate for the production of fibrinolytic enzyme. This result establishes the use of cuttle fish waste and cow dung mixture for fibrinolytic enzyme production. In enzyme bioprocess, no defined culture medium has been proposed to enhance the production of enzymes from various microbial sources. Every organism is unique in the requirement of its own environmental and nutritive factors for more enzyme production. Hence, optimization of enzyme production by an individual organism is a key to enhance enzyme production. These kinds of studies would provide low cost substrates for enzyme production in industries.

### Use of one variable at a time approach (OVAT) for screening medium components

**Fig. 2:**
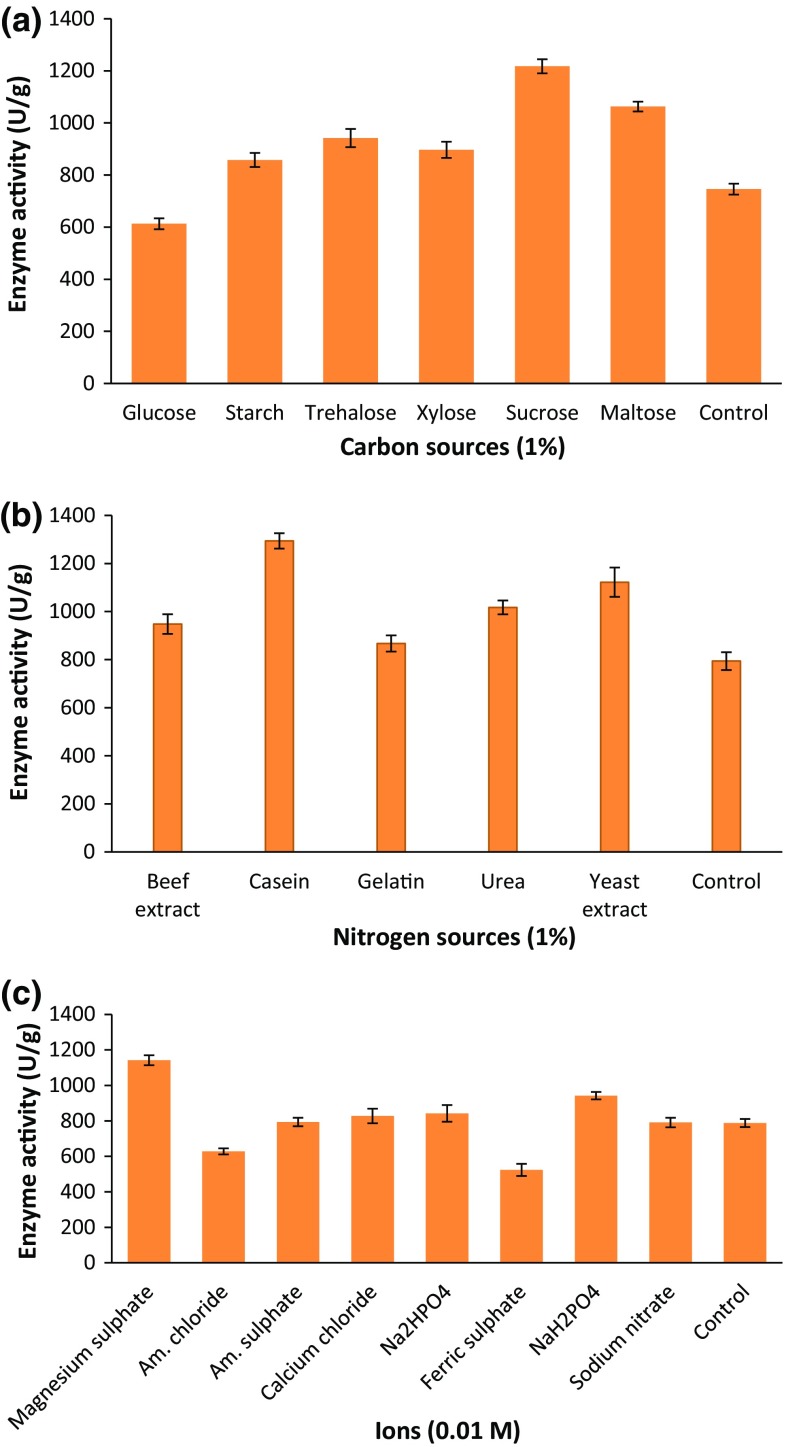
Effect of carbon source (**a**) nitrogen source (**b**) and ion (**c**) on fibrinolytic enzyme production

The traditional OVAT experiment helps to screen the variables without any complicated analysis. The mixture of cuttle fish waste and cow dung was used as the substrate for optimization of fibrinolytic enzyme production. In the present study, experiments were carried out with different carbon (glucose, starch, trehalose, xylose, sucrose, and maltose), nitrogen (beef extract, casein, gelatin, urea, and yeast extract) and ionic sources (ammonium chloride, magnesium sulfate, ammonium sulfate, calcium chloride, di-sodium hydrogen phosphate (Na_2_HPO_4_), sodium di-hydrogen phosphate (NaH_2_PO_4_), ferrous sulfate, and sodium nitrate) to evaluate the suitable nutrient source for the process of fibrinolytic enzyme production. All carbon sources, except glucose containing culture medium did not exhibit fibrinolytic activity than that of control and this indicated the inducible nature of *B*. *cereus* IND5 fibrinolytic enzyme. Among the carbon sources, sucrose stimulated more enzyme production (1218 ± 27 U/g) (Fig. 2a). Hence, sucrose was used as the suitable carbon source. Vijayaraghavan and Vincent (2014) concluded that sucrose was the best carbon source for fibrinolytic enzyme production for *Paenibacillus* sp. IND8. Among the nitrogen sources, casein considerably enhanced fibrinolytic enzyme production (1294 ± 32 U/g) (Fig. 2b). This result agreed with the observations made with *Bacillus* sp. strain AS-S20-I (Mukherjee and Rai 2011) and *Proteus penneri* SP-20 (Jhample et al. 2015). MgSO_4_ (0.1%, w/w) was found to be the best ionic source for production of fibrinolytic enzyme (1142 ± 28 U/g). However, addition of ammonium chloride (0.1%, w/w) negatively influenced the enzyme production (Fig. 2c). Based on OVAT approach, sucrose, casein and MgSO_4_ were chosen for further experiments.

### Medium optimization using 2^5^ full factorial design

In this study, cow dung and cuttle fish waste were (50:50) used for the production of enzyme. A two-level five factorial design is a best statistical tool for studying the production of fibrinolytic enzymes (Liu et al. 2005). This method analyzes the factors in two levels, i.e., high (+) and low (−), for the screening of important variables or factors (Table 1). From the results of preliminary screening carried out by OVAT approach, sucrose, casein and MgSO_4_ were selected as the suitable nutritional factors for evaluating fibrinolytic enzyme production. In this statistical design, along with these three variables, moisture and pH were also included as the critical factors for optimizing enzyme production in SSF. The variables and the results of the experiments were described in the Table 2. In this study, the production of fibrinolytic enzyme varied between 180 and 5044 U/g. The production of enzyme was observed to be maximum in the medium containing 80% moisture content, 0.1% sucrose, 0.1% casein, 0.1% MgSO_4_ and pH 7.0. ANOVA of the interactive effects were represented in the Table 3. The *F* value of this model was 128.47 and it was statistically significant. There is only a little chance (0.01%) that this much large “Model *F*-value” had occurred due to noise. Hence, the interactive tested variables were found to be statistically significant. The model terms A, B, D, E, AB, AC, AD, AE, BD, BE, CE, DE, ABC, ABE, ACD, ACE, ADE, BCD, BCE, BDE, ABCE, ACDE and BCDE were found to be significant. The predicted *R* ^2^ value (0.9263) was found to be close to the adjusted *R* ^2^ value (0.9911). The *R* ^2^ value of this model was 0.9988. The equation of the model can be written using coded levels of factors as:

**Table 1:** The independent variables chosen for 2^5^ factorial design and their coded levels

Symbol	Variables	Units	Coded levels	
			−1	+1
A	pH		7	9
B	Moisture	(%)	80	100
C	Sucrose	(%)	0.1	1.0
D	Casein	(%)	0.1	1.0
E	MgSO_4_	(%)	0.01	0.10

**Table 2:** Randomized runs of 2^5^ factorial design and the measured response

Run	A:pH	B:Moisture	C:Sucrose (%)	D:Casein (%)	E:MgSO_4_ (%)	Enzyme activity (U/g)
1	1	1	1	−1	1	1693
2	−1	−1	−1	1	1	2730
3	1	−1	1	1	1	1258
4	−1	1	1	1	−1	2019
5	1	−1	1	1	−1	2997
6	−1	−1	1	−1	1	2320
7	1	−1	−1	1	1	821
8	1	−1	1	−1	1	1166
9	1	1	1	−1	−1	2233
10	−1	1	−1	−1	−1	2310
11	1	−1	−1	−1	−1	530
12	1	1	−1	−1	−1	4251
13	1	−1	−1	−1	1	1546
14	−1	1	−1	−1	1	180
15	1	−1	−1	1	−1	2031
16	1	1	1	1	−1	2544
17	−1	−1	1	−1	−1	2754
18	−1	1	−1	1	1	1750
19	1	1	−1	−1	1	230
20	−1	−1	−1	−1	−1	3587
21	−1	1	1	1	1	3605
22	1	−1	1	−1	−1	3198
23	−1	1	−1	1	−1	910
24	1	1	−1	1	−1	4640
25	−1	−1	−1	−1	1	5044
26	−1	−1	1	1	−1	2521
27	−1	1	1	−1	1	2818
28	−1	−1	−1	1	−1	3651
29	1	1	−1	1	1	3840
30	−1	−1	1	1	1	3340
31	−1	1	1	−1	−1	2251
32	1	1	1	1	1	1510

**Table 3:** ANOVA results for 2^5^ factorial design

Source	Sum of squares	*df*	Mean square	*F* value	*p* value	
Model	4.876+007	27	1.806E+006	128.47	0.0001	Significant
A-pH	1.759E+006	1	1.759E+006	125.13	0.0004	
B-Moisture	2.646E+005	1	2.646E+005	18.83	0.0123	
C-Sucrose	18.00	1	18.00	1.281E−003	0.9732	
D-Casein	4.646E+005	1	4.646E+005	33.06	0.0045	
E-MgSO_4_	2.192E+006	1	2.192E+006	155.98	0.0002	
AB	9.788E+006	1	9.788E+006	696.37	<0.0001	
AC	2.042E+005	1	2.042E+005	14.52	0.0189	
AD	1.027E+006	1	1.027E+006	73.05	0.0010	
AE	4.762E+006	1	4.762E+006	338.77	<0.0001	
BC	41184.50	1	41184.50	2.93	0.1621	
BD	1.069E+006	1	1.069E+006	76.03	0.0010	
BE	2.258E+005	1	2.258E+005	16.06	0.0160	
CD	40186.13	1	40186.13	2.86	0.1661	
CE	2.384E+005	1	2.384E+005	16.96	0.0146	
DE	3.737E+005	1	3.737E+005	26.59	0.0067	
ABC	1.069E+007	1	1.069E+007	760.25	<0.0001	
ABE	1.474E+005	1	1.474E+005	10.49	0.0317	
ACD	2.193E+006	1	2.193E+006	156.05	0.0002	
ACE	3.659E+005	1	3.659E+005	26.03	0.0070	
ADE	1.093E+005	1	1.093E+005	7.77	0.0494	
BCD	1.065E+006	1	1.065E+006	75.77	0.0010	
BCE	3.523E+006	1	3.523E+006	250.65	<0.0001	
BDE	3.109E+006	1	3.109E+006	221.17	0.0001	
CDE	14620.50	1	14620.50	1.04	0.3654	
ABCE	2.869E+005	1	2.869E+005	20.41	0.0107	
ACDE	2.957E+005	1	2.957E+005	21.04	0.0101	
BCDE	4.512E+006	1	4.512E+006	321.00	<0.0001	
Residual	56223.75	4	14055.94			
Cor total	4.881E+007	31				

3 $$\begin{aligned} {\text{Fibrinolytic enzyme activity}} = &+ 2 3 8 9. 9 4- 2 3 4. 4 4 {\text{A}} - 90. 9 4 {\text{B}} \\ & - 0. 7 5 {\text{C}} + 1 20. 50{\text{D}} - 2 6 1. 7 5 {\text{E}} + 5 5 3.0 6 {\text{AB}} - 7 9. 8 7 {\text{AC}} + 1 7 9. 1 3 {\text{AD}} - 3 8 5. 7 5 {\text{AE}} \\ & + 3 5. 8 7 {\text{BC}} + 1 8 2. 7 5 {\text{BD}} - 8 4.00{\text{BE}} - 3 5. 4 4 {\text{CD}} + 8 6. 3 1 {\text{CE}} + 10 8.0 6 {\text{DE}} \\ & - 5 7 7. 8 8 {\text{ABC}} - 6 7. 8 7 {\text{ABE}}{-} 2 6 1. 8 1 {\text{ACD}}{-} 10 6. 9 4 {\text{ACE}} - 5 8. 4 4 {\text{ADE}} \\ & - 1 8 2. 4 4 {\text{BCD}} + 3 3 1. 8 1 {\text{BCE}} + 3 1 1. 6 9 {\text{BDE}} + 2 1. 3 7 {\text{CDE}} + 9 4. 6 9 {\text{ABCE}} \\ & - 9 6. 1 2 {\text{ACDE}}{-} 3 7 5. 50{\text{ BCDE}} .\\ \end{aligned}$$

The negative coefficients for medium components A (pH), B (moisture), C (sucrose) and E (MgSO_4_) indicated that the enzyme production can be increased by decreasing their concentrations in the fermentation medium. The positive coefficient for the model term D (casein) indicated that enzyme production could be increased by increasing the amount of casein in the SSF medium. Based on the *F* value from Table 3, the medium components casein, MgSO_4_ and pH were considered as the vital components for fibrinolytic enzyme production by *B*. *cereus* IND5 using cuttle fish waste and cow dung mixture.

### Optimized enzyme production SSF process using RSM

RSM is an ideal statistical tool for optimizing enzyme production. Recently, RSM was applied for optimized production of cellulases (Singh et al. 2014; Premalatha et al. 2015), fibrinolytic enzyme (Majumdar et al. 2015) and xylanase (Khusro et al. 2016). The optimum concentration of individual process parameters considering the interactive effect were elucidated using RSM. Hence, the vital parameters were taken at five levels and SSF was done according to CCD. The factors and their levels were described in Table 4. The enzyme activity was found to vary from 1488 to 5364 U/g (Table 5). ANOVA was used to evaluate the results and the model was statistically significant with *F* value of 1276.39 (Table 6). The chance of interplay of noise in the appearance of this much large *F* value is as little as 0.01%. The model terms A, B, C, AC, AB, BC, A^2^, B^2^ and C^2^ were significant. The “Lack of fit *F* value” of 1.02 implies the insignificance of lack of fit as compared to the pure error. There was an estimated chance of 48.97% that a “Lack of Fit-value” of this magnitude could occur owing to noise. “The Pred R-squared” of 0.9960 was close to the “Adj R-Squared” of 0.9983. The second order polynomial equation is given below.

**Table 4:** The independent variables selected for CCD and their coded values

Variables	Symbol	Coded values				
		−*α*	−1	0	+1	+*α*
pH	A	6.32	7	8	9	9.68
Casein	B	−0.21	0.10	0.55	1.00	1.31
MgSO_4_	C	0.01	0.03	0.05	0.07	0.09

**Table 5:** Central composite design runs and results

Run	A:pH	B:Casein	C:MgSO_4_	Enzyme activity (U/g)
1	−1.000	1.000	−1.000	5201
2	0.000	0.000	−1.682	4457
3	0.000	0.000	0.000	3421
4	1.000	−1.000	1.000	5364
5	0.000	−1.682	0.000	3682
6	0.000	1.682	0.000	3358
7	0.000	0.000	0.000	3425
8	−1.682	0.000	0.000	2126
9	0.000	0.000	0.000	3410
10	0.000	0.000	0.000	3512
11	1.000	1.000	−1.000	3528
12	0.000	0.000	0.000	3430
13	−1.000	−1.000	1.000	1488
14	0.000	0.000	0.000	3398
15	0.000	0.000	1.682	2569
16	1.000	−1.000	−1.000	4107
17	1.682	0.000	0.000	3852
18	1.000	1.000	1.000	2609
19	−1.000	1.000	1.000	1617
20	−1.000	−1.000	−1.000	2830

**Table 6:** ANOVA for CCD design results

Source	Sum of squares	*df*	Mean square	*F* value	*p* value	
Model	1.910E+007	9	2.122E+006	1276.39	<0.0001	Significant
A-pH	3.982E+006	1	3.982E+006	2395.44	<0.0001	
B-Casein	1.392E+005	1	1.392E+005	83.74	<0.0001	
C-MgSO_4_	4.413E+006	1	4.413E+006	2654.43	<0.0001	
AB	4.254E+006	1	4.254E+006	2559.06	<0.0001	
AC	3.464E+006	1	3.464E+006	2083.43	<0.0001	
BC	2.440E+006	1	2.440E+006	1467.57	<0.0001	
A^2^	3.471E+005	1	3.471E+005	208.77	<0.0001	
B^2^	15266.04	1	15266.04	9.18	0.0127	
C^2^	13032.65	1	13032.65	7.84	0.0188	
Residual	16625.01	10	1662.50			
Lack of fit	8413.68	5	1682.74	1.02	0.4897	Not significant
Pure error	8211.33	5	1642.27			
Cor total	1.911E+007	19				

4 $$\begin{aligned} {\text{Enzyme}}\;{\text{activity}} & = + 3 4 3 2. 4 1 + 5 40.0 1 {\text{A}} - 100. 9 7 {\text{B}}{-} 5 6 8. 4 5 {\text{C}} \\ & \quad {-} 7 2 9. 2 5 {\text{AB}} + 6 5 8.00{\text{AC}}{-} 5 5 2. 2 5 {\text{BC}}{-} 1 5 5. 1 9 {\text{A}}^{ 2} + 3 2. 5 5 {\text{B}}^{ 2} + 30.0 7 {\text{C}}^{ 2} . \\ \end{aligned}$$

**Fig. 3:**
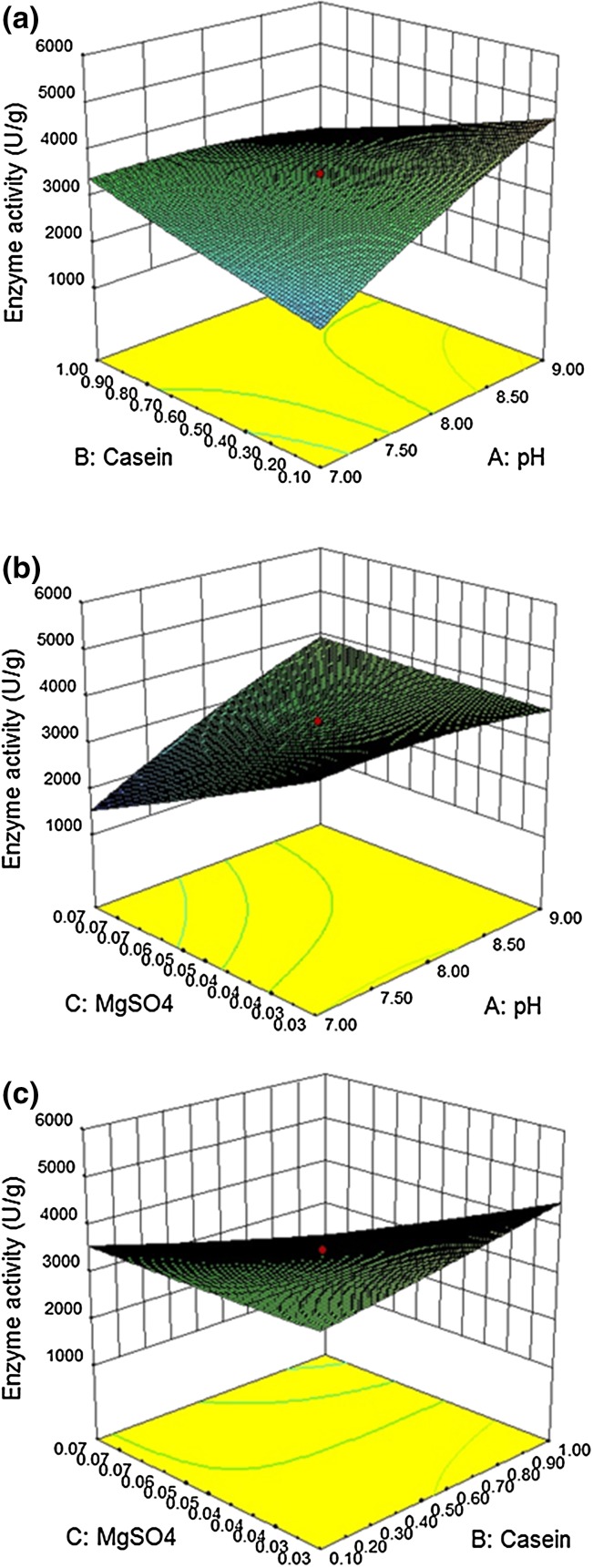
Three dimensional response surface plots showing the effect of (**a**) pH and casein, (**b**) pH and MgSO_4_ and (**c**) casein and MgSO_4_

To deduce the optimum concentration of each variable for the enhancement of enzyme production 3D surface plots were constructed using RSM (Fig. 3). The optimized values of pH, casein and MgSO_4_ were 7.8, 1.1 and 0.08%, respectively. To check the predicted response, a validation experiment was conducted. Under the optimum levels of medium components, the fibrinolytic enzyme production was observed to be 5247 ± 37 U/g. The predicted response 5201 U/g was close to the observed results and this validated the selected model. This finding shows the aptness of the selected model, indicating that the optimized levels of medium components favor the fibrinolytic enzyme production in SSF.

The culture medium cost is approximately 30–40% of total production cost of enzymes (Joo et al. 2003), hence the application of low cost substrate could reduce the overall enzyme cost. In the present observation, enzyme yield was found to be high in SSF than submerged fermentation. The processing cost of cow dung and cuttle fish waste was approximately 10 INR/kg material. The cost of one of the commercially available culture medium (Nutrient broth, HIMEDIA, Mumbai, India) was approximately 4 INR/g material. The approximate yield and production medium cost was 1400 U/INR in submerged fermentation, whereas 500,000 U/INR in SSF. The media cost was more than 100 times cheap in SSF than submerged fermentation. Thus, the use of cuttle fish waste and cow dung could reduce minimum 30% of overall enzyme cost.

### Downstream purification of the produced fibrinolytic enzyme

**Fig. 4:**
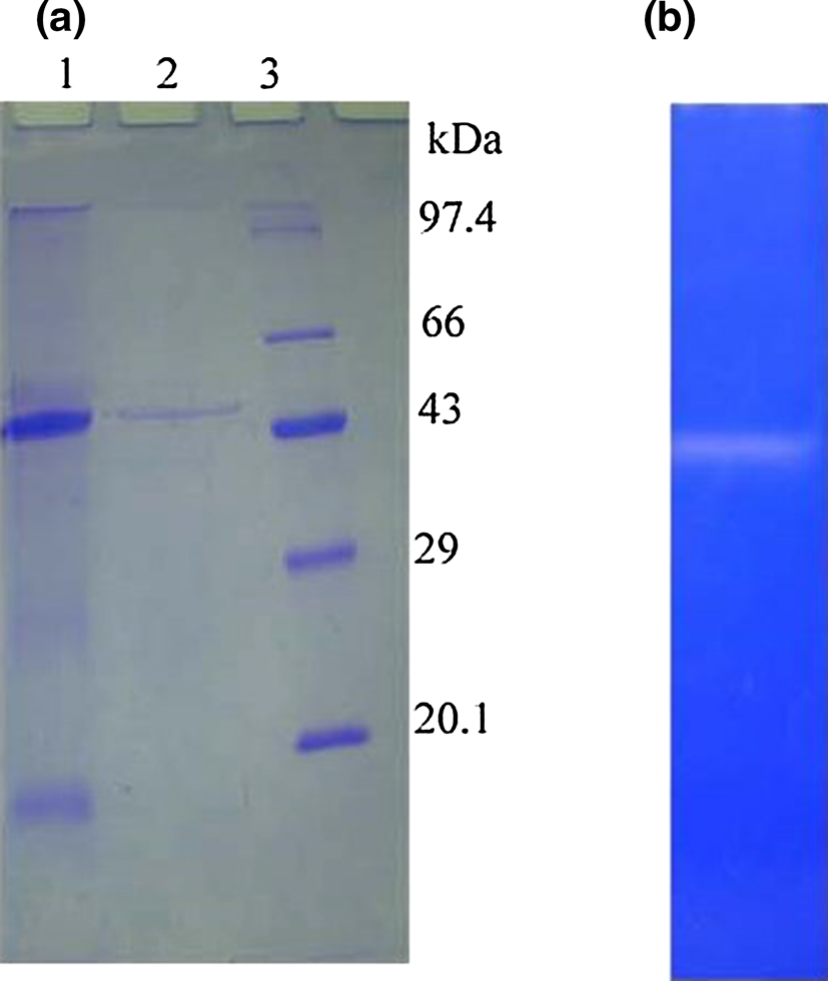
**a** SDS–polyacrylamide gel electrophoresis (12%) after coomassie staining. *lane 1*–DEAE-cellulose fraction; *lane 2*–affinity chromatography purified enzyme; *lane 3*–molecular markers. **b** Fibrin zymography showing the purified fibrinolytic enzyme from *B. cereus* IND5

In this study, fibrinolytic enzyme was purified by the combination of ammonium sulfate precipitation, DEAE-cellulose column chromatography and affinity chromatography using casein–agarose matrix. The ammonium sulfate precipitation showed 22,040 U of fibrinolytic enzyme with 43% yield, and 1.3-fold purification was achieved. The specific activity of fibrinolytic enzyme was increased to 143 U/mg protein after DEAE-cellulose chromatography purification. The affinity chromatography purified fractions showed 364.5 U/mg protein. The highly active fraction from DEAE cellulose and casein-agarose chromatography fractions was analyzed for its homogeneity. The DEAE chromatography fractions showed partial purification of enzyme. The affinity purified enzyme migrated as a single band in SDS-PAGE gel and the molecular weight was found to be 47 kDa (Fig. 4). The molecular weight of bacterial fibrinolytic enzyme varies widely. In *B*. *subtilis* KCK-7, the molecular weight was registered as 44 kDa (Paik et al. 2004); however, the molecular weight of fibrinolytic enzyme from *B. subtilis* LD-8547 was reported as 30 kDa (Wang et al. 2008a).

### Characterization of the produced fibrinolytic enzyme

**Fig. 5:**
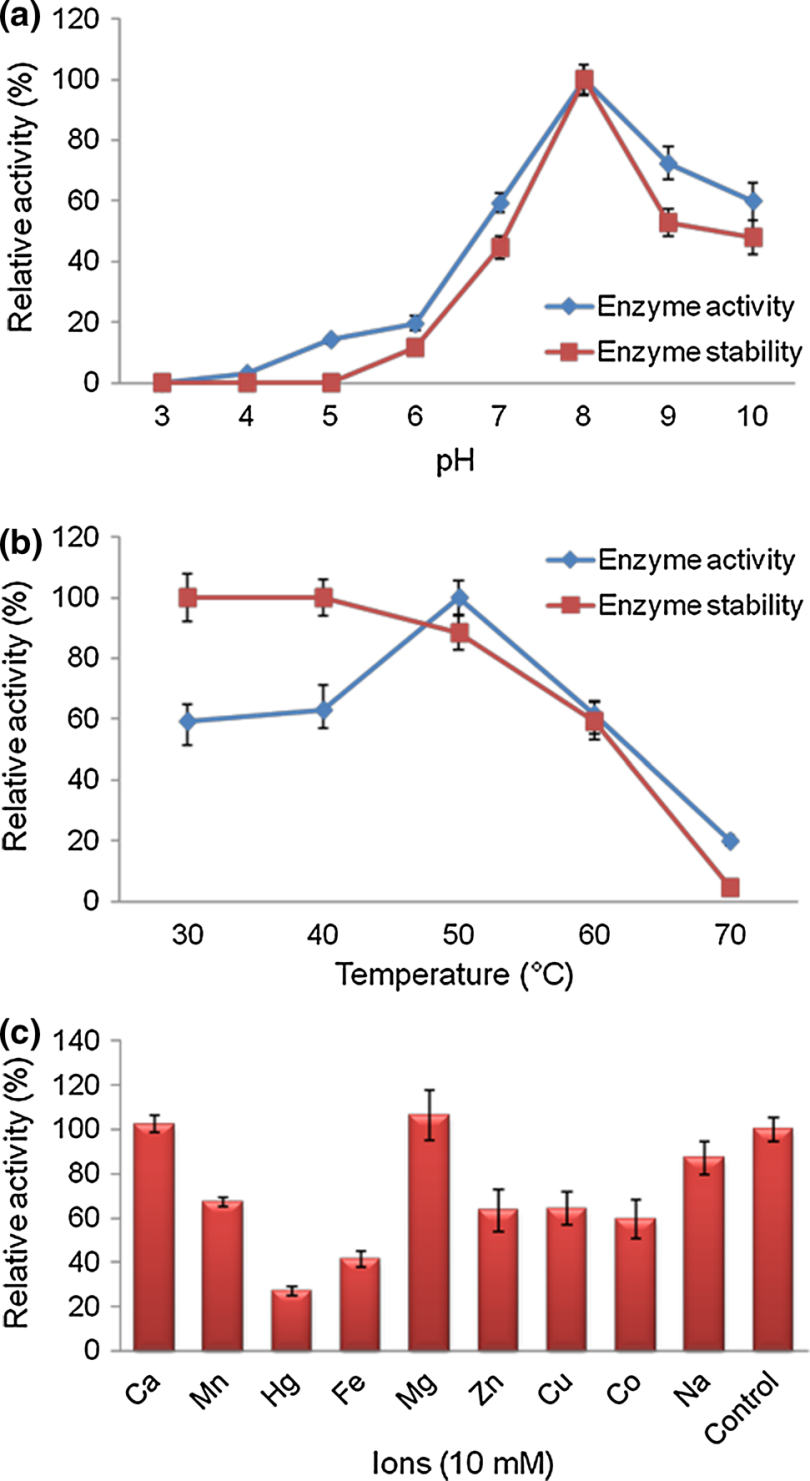
Biochemical characterization of the purified fibrinolytic enzyme. **a** Effect of pH on enzyme activity and stability. Enzyme assay was carried out at 37 °C. **b** Effect of temperature on enzyme activity and stability. Enzyme assay was carried out at pH 8.0 with 0.1 M Tris buffer. **c** Effect of various ions on enzyme activity. Enzyme assay was carried out at pH 8.0 and at 50 °C

The characterization of the purified enzyme was done by analyzing the effect of temperature, pH, and ionic strength on its stability and activity. Figure 5a revealed the effect of pH on enzyme activity and stability. *B. cereus* IND5 fibrinolytic enzyme showed maximum activity at pH 8.0 and was stable in the range of pH from 7.0 to 9.0 which throws light on the possible applications of this enzyme in a plethora of industrial sectors. This result was acceptable with respect to the previously published results. The fibrinolytic enzyme isolated from various *Bacillus* sp. showed optimum activity in the range, pH 8.0–9.0 (Agrebi et al. 2010; Mahajan et al. 2012; Bajaj et al. 2013). *B. cereus* IND5 fibrinolytic enzyme showed maximum activity at 50 °C (Fig. 5b). Bajaj et al. (2013) reported that several *Bacillus* sp. fibrinolytic enzymes have shown optimum activity at 35–40 °C. However, 60 °C was optimum for the activity of fibrinolytic enzyme produced from *B. subtilis* A26 (Agrebi et al. 2009). *B. cereus* IND5 fibrinolytic enzyme activity was inhibited by the presence of tested metal ions except Mg^2+^ and Ca^2+^ (Fig. 5c). It was previously reported that the fibrinolytic enzyme of *B. subtilis* A26 was activated in the presence of Ba^2+^, Cu^2+^, K^+^, Mg^2+^, Mn^2+^ and Na^+^ and enzyme activity was inhibited by the addition of Zn^2+^ and Hg^2+^ (Agrebi et al. 2009). In *B. subtilis* ICTF-1, fibrinolytic enzyme activity was inhibited by ions like Zn^2+^, Hg^2+^ and Fe^3+^ (Mahajan et al. 2012).

## Conclusion

The present study revealed that *B*. *cereus* IND5 effectively utilized cuttle fish waste and cow dung for its growth and enzyme production. This substrate could have unprecedented potential for the production of fibrinolytic enzyme. The interactions of pH, casein, and magnesium sulfate were evaluated by response surface methodology. Fibrinolytic enzyme production was significantly increased by altering the medium pH and at increased concentration of casein. The purified enzyme was highly active at 50 °C and was stable up to pH 8.0. Considering these properties, it could be useful for various biotechnological applications.
